# The Early Hematological Profile and Its Variations: A Useful Tool in the Prediction of Intraventricular Hemorrhage in Extremely Preterm Infants

**DOI:** 10.3390/medicina60030410

**Published:** 2024-02-28

**Authors:** Manuela Cucerea, Mihaela Moscalu, Marta Simon, Maria Livia Ognean, Melinda-Ildiko Mitranovici, Diana Maria Chiorean, Raluca Marian

**Affiliations:** 1 Department of Neonatology, George Emil Palade University of Medicine, Pharmacy, Science, and Technology, 540142 Targu Mures, Romania; manuela.cucerea@umfst.ro (M.C.); marta.simon@umfst.ro (M.S.); 2 Department of Preventive Medicine and Interdisciplinarity, Grigore T. Popa University of Medicine and Pharmacy, 700115 Iași, Romania; 3 Dental Medicine and Nursing Department, Faculty of Medicine, Lucian Blaga University of Sibiu, 550169 Sibiu, Romania; maria.ognean@ulbsibiu.ro; 4 Department of Obstetrics and Gynecology, Emergency County Hospital Hunedoara, 331057 Hunedoara, Romania; mitranovicimelinda@yahoo.ro; 5 Department of Pathology, County Clinical Hospital of Targu Mures, 540072 Targu Mures, Romania; chioreandianamaria@yahoo.com; 6 Department of Pathophysiology, George Emil Palade University of Medicine, Pharmacy, Science and Technology, 540142 Targu Mures, Romania; 7 Department Cellular and Molecular Biology, George Emil Palade University of Medicine, Pharmacy, Science, and Technology, 540142 Targu-Mures, Romania; raluca.marian@umfst.ro

**Keywords:** intraventricular hemorrhage, extremely preterm infants, hematological parameters

## Abstract

*Background and Objectives*: The purpose of this study to investigate if the early variations in the hematological profile could be a useful tool in the prediction and evaluation of intraventricular hemorrhage. *Materials and Methods*: It is a retrospective study conducted between 1 January 2017 and 31 December 2022, in a tertiary academic center. In-born infants ≤ 28 weeks of gestation (*n* = 134) were enrolled. The study group of infants with all grades of IVH was further divided into mild IVH subgroups (grades 1 and 2) and severe IVH subgroups (grades 3 and 4); the control group included infants without IVH. *Results*: The prevalence of IVH was 35.8% (*n* = 48 of 134 infants—study group). We identified significantly lower median values of HGB (*p* = 0.0312) and HCT (*p* = 0.0172) in all grades of the IVH group at birth as compared with control, followed by a significantly higher drop in MCV (*p* = 0.0146) and MCH (*p* = 0.0002) in the fourth day of life. *Conclusions*: Extremely preterm infants with IVH may have lower HTC and HGB values at birth, together with a decrease in MCH and MCHC and increase in MPV. The predictive model based on logistic regression analysis could predict the probability of the occurrence of IVH according to their values.

## 1. Introduction

Intraventricular hemorrhage (IVH) is one of the most common life-threatening early complications of prematurity, particularly in infants born with a gestational age (GA) of 28 weeks or less. The incidence of IVH in extremely premature (EP) infants has been reported to be as high as 45%, leading to increased morbidity, mortality, and neurodevelopmental problems [1,2,3]. Most cases of IVH (50–90%) occur within the first 72 h of life (known as early IVH) [4,5,6]. The pathogenesis of IVH is multifactorial and involves rapid fluctuations in cerebral blood flow (CBF) as well as innate fragility of the germinal matrix [7].

The complete blood count (CBC) is an essential part of the diagnostic assessment for newborn infants. While many studies have reported on the changes in hematological parameters during the neonatal period, few of them have focused on EP, who are the most vulnerable infants [8,9,10,11]. Due to the immaturity and severity of complications, individuals in this high-risk population are vulnerable to hemorrhagic accidents, sepsis, and iatrogenic blood loss. These can significantly impact hematological values and clinical outcomes. Hematocrit (HCT) at birth can be used as a marker for intravascular volume status [12,13], while the hemoglobin (HGB) level is a guide for liberal or restrictive red blood cell (RBC) transfusion decisions [14,15]. Several studies have focused on the correlation between Hct levels and IVH incidence. A low Hct level at birth in EP infants can significantly increase cerebral blood flow (CBF) to compensate for cerebral hypoperfusion and low cerebral oxygenation, thereby leading to IVH [12,13].

Delayed cord clamping (DCC) has been found to improve HCT and HGB levels, reducing the incidence of significant IVH and the need for blood transfusion [16].

The main objective of this study was to analyze the correlation between blood parameters at birth and the occurrence of IVH in a group of EP infants. The secondary objective was to identify the neonatal risk factors associated with IVH.

## 2. Materials and Methods

A retrospective case-control single-center study was conducted at a tertiary referral center (Clinical County Emergency Hospital, Targu Mureș, Romania) from 1 January 2017 to 31 December 2022. The study aimed to evaluate the changes in hematological parameters associated with IVH in EP infants during the first four days of life. Clinical and laboratory data were analyzed.

### 2.1. Study Group: Inclusion and Exclusion Criteria

**Figure 1:**
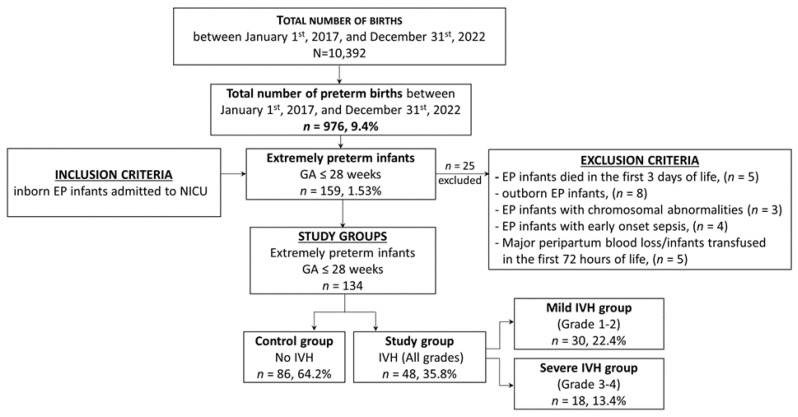
Selection of the study groups—flow chart.

A total of 159 preterm infants (1.53% of 10,392 births) born between 22–28 weeks of gestation (inclusion criteria) were admitted to the neonatal intensive care unit (NICU) during the study period. The exclusion criteria were as follows: outborn EP infants, death within the first three days of life, infants with chromosomal abnormalities, or with a history that could influence blood counts in the first days of life (e.g., significant peripartum blood loss, early-onset sepsis, and red blood cell (RBC) transfusion in the first 72 h). Therefore, 134 EP infants of 159 were enrolled in this study (Figure 1).

The study group consisted of infants born extremely preterm with all grades of IVH, which was further divided into two subgroups: the *mild IVH group* (those with grades 1 and 2) and *severe IVH group* (those with grades 3 and 4). EP infants without IVH were assigned to the control group. This classification allowed for a comparison of the hematological profile of infants with IVH irrespective of the severity of the condition, as well as a comparison of the profiles of those with mild and severe IVH.

We compared neonatal data between two groups, the study group (with any grade of IVH) and the control group (without IVH), as well as between two subgroups within the study group (mild IVH and severe IVH).

### 2.2. Data Collection

Clinical and laboratory data were collected by the research team over six years.

The patient underwent clinical and ultrasound evaluations, as well as blood sample collection, following the NICU protocol for the diagnosis and treatment of IVH. These assessments are part of the standard clinical procedure for managing EP infants. The mother provided written consent for this study. The GA was assessed using gestational ultrasound measurements and the new Ballard scoring system [17].

The medical record of each eligible EP infant was reviewed. Relevant data of prenatal and perinatal variables were extracted: gender, GA (in weeks), birth weight (BW, in grams), antenatal corticosteroids therapy (ACT: four doses of 6 mg dexamethasone every 12 h), mode of delivery (vaginal delivery vs. cesarean section), APGAR score (at 1 and 5 min), cord blood gases (pH, BE, lactate), presence and severity of respiratory distress syndrome RDS (mild, moderate, and severe), surfactant administration, nasal continuous positive airway pressure (nCPAP) support, invasive respiratory support (ventilation mode), presence of hypercapnia in the first days of life (pCO_2_ > 55 mmHg), hypotension (defined as a mean arterial blood pressure less than the infant’s GA, or < 30 mmHg), inotropic medication exposure (dopamine, dobutamine, and milrinone), patent ductus arteriosus (PDA), a need for RBC transfusion in days 4–7 after birth, NICU hospitalization (days), age at discharge (days), and survival. The delayed cord clamping (DCC—after at least 30–60 s) protocol was not applied to infants who were unstable at birth and required immediate extensive resuscitation. Physiologic-based cord clamping (PBCC—sectioning the umbilical cord after the cessation of pulsations) or immediate cord clamping (ICC—under 30 s) protocols were applied at birth to EP infants.

### 2.3. Clinical Suspicion of IVH

Although most IVH is clinically silent, the diagnosis was suspected in EP infants with subtle changes in the level of consciousness and tone, abnormal eye and limb movements, the presence of seizures, cardiorespiratory instability, or sudden clinical deterioration [18].

### 2.4. Cranial Ultrasound (CUS)

Intraventricular hemorrhage was diagnosed using standard cranial ultrasound with 7.5–12 MHz transducers of a LOGIQ e9 ultrasound machine (General Electric Medical Systems Co.’s, Bucuresti, Romania). Trained neonatologists routinely performed the scan. According to the NICU protocol, the initial CUS examination was performed immediately after stabilization to rule out prenatal IVH. Serial CUS exams were performed daily for the first week of life, followed by weekly exams until discharge or as needed. Intraventricular hemorrhage was classified by the modified Papile’s grading system using cranial ultrasonography. Grade 1: Hemorrhage restricted to the germinal matrix. Grade 2: Hemorrhage in the germinal matrix and extended to the ventricles without dilatation of the ventricles. Grade 3: Hemorrhage in the germinal matrix and extended to the ventricles with dilatation of the ventricles. Grade 4: Intraventricular hemorrhage with parenchymal hemorrhage [19,20,21].

### 2.5. Blood Sample Collection (Laboratory Data)

#### 2.5.1. Blood Gases

According to the hospital’s protocol, blood gases were collected from each patient’s umbilical cord artery at birth. We recorded the pH, base excess (BE), and lactate levels. Afterward, arterial blood gases were collected as required. We defined hypercapnia as pCO_2_ ≥ 60 mmHg, where pCO_2_ between 55–60 mmHg was considered permissive hypercapnia [22].

#### 2.5.2. Complete Blood Count

All patients must undergo a CBC test on admission to the NICU as per hospital protocol. The test is performed within the first hour of birth for EP infants born in the hospital. The following CBC is collected on the fourth day of life or as needed to minimize iatrogenic blood loss. The study analyzed both initial and fourth-day CBC values.

Blood samples (1 mL) were collected from central or peripheral venous blood before any erythrocyte transfusions and were processed using the same automated analyzer (Sysmex XT-4000). The following blood parameters were recorded: erythrocyte count (×10^6^/µL), HGB concentration (g/dL), HCT (%), mean corpuscular volume (MCV, µm^3^), mean corpuscular hemoglobin (MCH, pg), mean corpuscular hemoglobin concentration (MCHC, mg/dL), total leukocyte count (WBC, ×10^3^/µL), differential leukocyte count (×10^3^/µL), neutrophil count (×10^3^/µL), platelet count (PLT, ×10^3^/µL), mean platelet volume (MPV, µm^3^), and plateletcrit PCT (%).

### 2.6. Ethics Approval

The study was approved by the medical ethics committee of Targu Mures County Emergency Hospital, Romania (No. Ad. 35519/13.12.2019).

### 2.7. Statistical Analysis

The statistical analysis was performed using SPSS version 29 (Inc., Chicago, IL, USA) for Windows. Descriptive statistics such as means, medians, quartiles, and standard deviations were used to describe the study groups’ baseline, demographic, clinical characteristics, and hematological parameters. Continuous variables were presented as the mean (standard deviation—SD) if the distribution was normal and median and quartiles if the variable did not have a normal distribution. Categorical data (qualitative variables) were compared using Pearson’s Chi-square and Fisher’s exact tests. The independent t-test was used to analyze continuous data for comparisons. The odds ratio (OR) and 95% confidence interval (CI) were calculated.

Both univariate and multivariate analyses of the parameters were performed to evaluate the predictive neonatal factors for IVH. Statistical significance was defined as a *p*-value < 0.05.

## 3. Results

**Figure 2:**
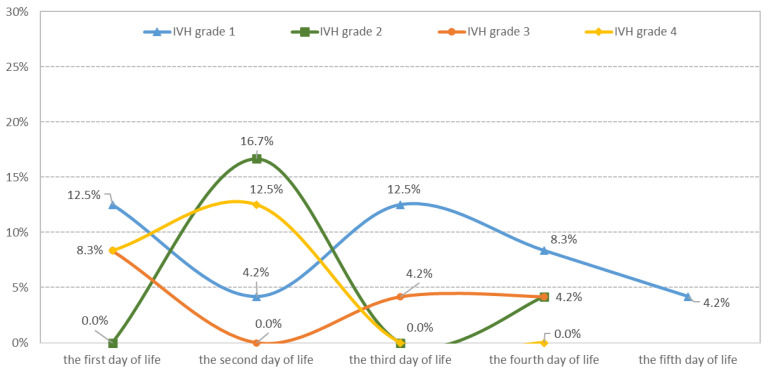
Distribution of IVH cases according to severity and the day of appearance.

The prevalence of IVH in EP infants was 35.82%, with 48 out of 134 infants affected. Most IVH cases occurred in the first three days of life (DOL), accounting for 79.2% of cases, while 20.8% of IVH cases occurred between the fourth and fifth DOL (Table 1 and Figure 2).

The baseline, demographic, and clinical characteristics of EP infants were compared based on IVH severity. The median values for GA and BW were comparable between the study group and the control group, even though the median BW was slightly lower in the study group. However, the severe IVH group had significantly lower BW median values than the mild IVH group (*p* = 0.019), as shown in Table 2.

A significant correlation has been found between the IVH group (all grades) and several factors like male gender (*p* = 0.004), lack of antenatal corticosteroid therapy (*p* = 0.041), vaginal delivery (*p* = 0.001), low Apgar score at 1 min (*p* = 0.043), lack of delayed cord clamping (DCC; *p* = 0.0023), immediate cord clamping (ICC; *p* = 0.0267), moderate and severe respiratory distress syndrome (RDS; *p* = 0.014; *p* = 0.003), the requirement of mechanical ventilation (MV; *p* = 0.006) and high-frequency oscillatory ventilation (HFOV; *p* = 0.014), hypercapnia (*p* < 0.001), hypotension with inotropic support in the first 4 days of life (*p* < 0.001), and the need for red blood cell transfusion (RCT) between days 4–7 of life (*p* < 0.001). There was no significant increase in the prevalence of IVH in infants who were born small for gestational age (SGA), had low Apgar scores at 5 min, received exogenous surfactant, or had PDA. Moreover, the IVH group (all grades) and controls had comparable NICU days of hospitalization.

In comparing EP infants with mild IVH to those with severe IVH, we found several factors that were significantly associated with severe IVH. These factors included a lack of antenatal corticosteroid therapy (*p* = 0.002), lower Apgar scores at 1 and 5 min (*p* = 0.007; *p* = 0.004), ICC (*p* = 0.001), moderate and severe RDS (*p* = 0.021; *p* = 0.025), hypercapnia (*p* < 0.001), and hypotension requiring inotropic support in the first four days of life (*p* < 0.001). In addition, infants with severe IVH were more likely to have been exposed to mechanical ventilation (MV) and high-frequency oscillatory ventilation (HFOV). However, there were no significant differences between the two groups regarding SGA, vaginal delivery, surfactant administration, and the presence of PDA (Table 2).

The median day of discharge was significantly lower in the IVH group (all grades) compared to the control group (36 vs. 50; *p* = 0.008). Additionally, patients with severe IVH had a shorter median length of stay in the hospital compared to those with mild IVH (23 vs. 52, *p* = 0.011), which may be due to higher mortality rates. Compared to the control group, mortality was significantly higher in the presence of any grade of IVH (4.7% vs. 20.8%; *p* = 0.003). The severe IVH subgroup had a higher mortality rate than the mild IVH subgroup (6.7% vs. 44.4%; *p* = 0.001) (Table 2).

We compared the cord blood gases and changes in hematological parameters between the IVH group (all grades) and the control group from birth to DOL4. We also compared the hematological parameters between different grades of IVH severity within the study group, specifically mild IVH versus severe IVH subgroups, as shown in Table 3.

### 3.1. Blood Gases at Birth

According to the research findings, the IVH group (all grades) had significantly lower pH levels (median value: 7.21 vs. 7.28; *p* = 0.0008), higher BE levels (median value: −8.15 mmol/L vs. −4.9 mmol/L; *p* = 0.0066), and higher lactate levels (median value: 4.7 mmol/L vs. 2.6 mmol/L; *p* = 0.0003) than the control group. These factors were found to be significantly associated with the occurrence of any grade of IVH. Severe IVH was particularly associated with lower pH (median value: 7.15 vs. 7.26; *p* = 0.0072), higher base excess (median value: −10.6 mmol/L vs. −6 mmol/L; *p* = 0.0032), and higher lactate levels (median value: 5.3 mmol/L vs. 3 mmol/L; *p* = 0.0187) when comparing mild and severe IVH subgroups.

### 3.2. Red Blood Cell Lineage

- Comparison between EP infants without IVH and those with IVH, irrespective of severity

On the day of birth (DOL1), the IVH group (all grades) had significantly lower median values of HGB (14.1 g/dL vs. 15.9 g/dL; *p* = 0.0312), HCT (42.8% vs. 51.2%; *p* = 0.0172), and ERY (3.99 × 10^6^ vs. 4.91 × 10^6^; *p* = 0.0251) when compared to the control group. On DOL4, the IVH group had a significant decrease in the median values of HGB (11.7 g/dL vs. 14.2 g/dL; *p* < 0.001), HCT (34.3% vs. 42.1%; *p* < 0.001), and ERY (3.02 × 10^6^/µL vs. 3.83 × 10^6^/µL; *p* < 0.001) when compared to the controls.

Regarding erythrocytes indices, on DOL1, the median MCV (mean corpuscular volume, μm^3^), MCH (mean corpuscular hemoglobin, pg), and MCHC (mean corpuscular hemoglobin concentration g/dL) did not show any significant differences between the IVH group and control group. However, MCHC was slightly lower in the IVH group. On the other hand, on DOL4, we found significant differences between MCV (102.9 μm^3^ vs. 105.4 μm^3^; *p* = 0.0278) and MCH (34.2 pg vs. 36.7 pg; *p* < 0.001) parameters in the IVH group. The group with IVH also showed a significantly higher drop in MCV (8.35 µm^3^ vs. 7.5 µm^3^; *p* = 0.0146) and MCH (3.7 pg vs. 1.4 pg; *p* = 0.0002) compared to the control group.

- Comparison between preterm infants with mild IVH and those with severe IVH

At birth (DOL 1), there were no significant differences in HGB, HCT, and ERY count between mild and severe IVH subgroups but lower median values were observed in the severe IVH subgroup. Significant changes were noted on DOL4. Infants with severe IVH had significantly lower median levels of HGB (7.3 g/dL vs. 12.4 g/dL; *p* < 0.001), HCT (28.5% vs. 36.4%; *p* < 0.001), and ERY (2.73 × 10^6^/µL vs. 3.09 × 10^6^/µL; *p* = 0.0093) than infants with mild IVH.

Additionally, infants from the severe IVH subgroup exhibited a significant reduction in HGB levels (5.8 g/dL vs. 2.3 g/dL; *p* = 0.0029), HCT levels (9.9% vs. 8.1%; *p* = 0.0239), and ERY count (1.11 × 106 vs. 0.92 × 106; *p* = 0.0216).

The severe IVH subgroup on DOL1 exhibited a significantly lower MCH (35.6 pg vs. 39.2 pg; *p* < 0.001) and MCHC (32.4 g/dL vs. 34.3 g/dL; *p* = 0.0041) compared to the mild IVH subgroup and this difference remained consistent on DOL4 as well.

### 3.3. White Blood Cell Lineage

No significant differences were found between the IVH group (all grades) and the control group in terms of the median number of leukocytes and absolute neutrophil count (ANC), neither in DOL1 nor in DOL4. However, ANC was significantly lower in the severe IVH subgroup than the mild IVH subgroup in both DOL1 and DOL4. Only the I/T ratio was significantly lower in DOLI in the IVH group (all grades) compared to the controls (0.14 vs 0.18; *p* = 0.0007).

### 3.4. Platelet Lineage

There were no significant differences in median PLT, MPV, or PCT between the IVH group and the control group, as well as between the severe IVH subgroup and the mild IVH subgroup on DOL1. However, on DOL4, median PLT significantly decreased in the IVH group (201 × 10^3^/µL vs. 258 × 10^3^/µL; *p* = 0.0098) when compared to the control group and in the severe IVH subgroup (166 × 10^3^/µL vs. 256 × 10^3^/µL; *p* = 0.0106) when compared to the mild IVH subgroup.

The differences between DOL1 and DOL4 regarding platelet count were statistically significant in the IVH group (*p* = 0.0004) vs. controls but not between the severe IVH subgroup vs. the mild IVH subgroup. The median MPV (11.3 μm^3^ vs. 10.7 μm^3^; *p* = 0.0009) was significantly higher in the IVH group compared to controls on DOL4.

### 3.5. Univariate Analysis for AUC Evaluation of Hematological Parameters and Other Neonatal Factors Associated with IVH Risk in Extremely Preterm Infants

**Figure 3:**
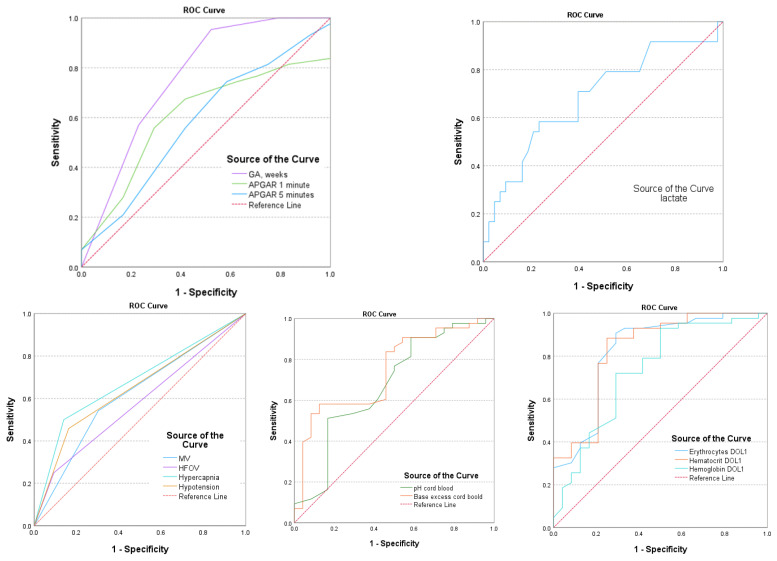
AUC—area under the curve for postnatal parameters associated with IVH.

Intraventricular hemorrhage in EP infants has a multifactorial determination. The research analyzed multiple factors including GA, Apgar scores after 1 and 5 min of birth, cord pH, BE, lactate levels at birth, pCO_2_ exceeding 55 mmHg (hypercapnia), treated hypotension, the requirement for MV and HFOV, HGB, HTC levels, and ERY count on the first day after birth. The aim was to determine the potential of these factors in predicting the occurrence of IVH. The findings are presented in Table 4 and Figure 3.

The gestational age and MV are strong predictors of IVH, with AUC values of 0.760 (95% CI: 0.669–0.851; *p* = 0.001) and 0.620 (95% CI: 0.519–0.720; *p* = 0.022), respectively. Hypercapnia and hypotension also have predictive IVH values, with AUC values of 0.680 (95% CI: 0.581–0.780; *p* = 0.001) and 0.648 (95% CI: 0.547–0.749; *p* = 0.05), respectively. Additionally, hematological parameters such as HGB, HCT, and ERY levels in DOL1 were found to be significant predictors (*p* < 0.005) for IVH, with AUC values of 0.806 (95% CI: 0.841–0.985), 0.922 (95% CI: 0.841–0.985), and 0.895 (95% CI: 0.796–0.963), respectively (as shown in Table 4 and Figure 3).

If the HGB level is below 13.9 g/dL, it is highly likely that IVH may occur, with a sensitivity of 89.6% and specificity of 88.6%. Similarly, if the HCT level is lower than 42.6% or the ERY count is less than 3.82 × 10^6^, the chances of IVH significantly increase, with a sensitivity of 88.4% and 89.6% and specificity of 91.3% and 90.4%, respectively.

### 3.6. Multivariate Analysis to Evaluate the Chance of IVH Occurrence

Independent variables for this analysis were selected based on the results of univariate analysis for AUC evaluation. Variables with a significance level lower than 0.05 (*p*  <  0.05) in the univariate analysis (Table 5) were considered as independent variables: GA, MV, hypercapnia, hypotension, pH, BE, cord lactate, and initial HCT (allocation of variables in Table 5). The regression process adopted the gradual regression method (backward stepwise—Wald) by which the variables for which the *p*-values were higher than 0.05 were excluded. Multivariate analysis by logistic regression allowed the assessment of the probability of IVH occurrence based on the corresponding β coefficients (Table 5). The prediction model expression was

$$p=\frac{1}{1+e^{-\left(constant+\beta 1\times GA+\beta 2\times {pCO}_{2}+\beta 3\times hypotension+\beta 4\times cord\;BE+\beta 5\times Hct\right)}}$$

Based on this analysis, a profile of the EP infant with IVH can be created. Multiple logistic regression analysis revealed that IVH is associated with several factors, including lower GA and cord BE, lower initial HCT, hypercapnia, and hypotension that requires treatment during the first few days of life.

## 4. Discussion

In our study, IVH prevalence (all grades) was 35.8% and severe IVH occurred in 13.4% of the EP patients. Most cases of IVH were diagnosed within the first three days of life. A 2021 literature review found global IVH incidence ranging from 7–72% for all grades and 6–22% for severe IVH [23]. The survival rate of EP infants in our study was 89.55%, higher than in other reports (62.3–89%) [24,25,26,27]. However, it must be interpreted considering that infants with early sepsis, congenital malformations, and deaths in the first three days of life were excluded from the initial cohort.

For patients with GA of 28 weeks or less, the HCT and HGB levels in the first few hours after delivery are lower compared to those in late preterm and term neonates. HCT is about 10 points lower and the HGB value is 3.3 g/dL lower [8]. In our study, the median HGB value at birth was 14.1 g/dL in the IVH group, 14.7 g/dL in the mild IVH subgroup, and 13.1 g/dL in the severe IVH subgroup. Initial HCT and ERY counts were also significantly lower in the IVH group than in controls. HGB levels lower than 13.9 g/dL and HCT levels lower than 42.6% significantly increased the chance of IVH in our study. Similarly, Hosono and colleagues reported that premature infants with an HGB level below 15 g/dL at birth had a higher incidence of IVH [28]. Another study conducted on extremely low birth weight infants showed that if the initial HCT level is lower than 45%, the risk of IVH is more than doubled (OR = 2.38, 95% CI: 1.19–4.76) [13].

On the other side, the EP infants with low initial HGB and HCT values had lower Apgar scores and required more extensive resuscitation measures during stabilization. Consequently, only 6.3% of IVH group patients were able to receive delayed umbilical cord clamping and placental transfusion. It is possible that this situation could be the reason behind the lower hematological values that were observed in this group.

The median values of initial HGB (15.9 g/dL), HCT (51.2%), and ERY (4.91 × 106) were found to be higher in EP infants without IVH but 23.3% of these infants benefited from delayed cord clamping. In a study conducted by Fogarty et al., it was reported that DCC increased the peak of HCT by 2.73 percentage points (95% CI: 1.94–3.52; *p* < 0.001) [29]. Another study conducted by Strauss et al. found that DCC led to an increased circulating RBC volume/mass (*p* = 0.04) and higher weekly Hct values (*p* < 0.005) [30].

The HGB, HCT, and ERY values may fluctuate after birth due to physiological changes, blood loss, and postnatal diseases, leading to anemia and a need for RBC transfusions in EP infants [31,32]. All EP infants in our study showed a statistically significant decline in HGB, HCT, and ERY count between the first and fourth day of life, especially for infants who developed IVH and severe IVH. The decline in HGB was 1.7 g/dL in the control group, 2.45 g/dL in all IVH group, 5.8 g/dL in the mild IVH subgroup, and 2.3 g/dL in the severe IVH group. The HCT decreased by 6.1% in the control group, 8.6% in all IVH group, 8.1% in the mild IVH subgroup, and 9.9% in the severe IVH subgroup. A previous study by Jopling found that infants with a gestational age of less than 29 weeks experienced a 6.0 ± 0.3% decrease in HGB and HCT values in the first 4 h after birth. A linear reduction in HCT and HGB values was observed in the first four weeks of life [33].

There is limited information available about erythrocyte indices in EP infants. Directly-measured MCV estimates the average size (volume) of circulating erythrocytes. The normal range for red blood cell size in adults is between 80–100 µm^3^ (fL). The size of red blood cells in newborns, especially preterm infants, is larger (MCV > 110 fL) than in adults due to increased fetal erythropoietic activity in response to intrauterine hypoxia. As a result, many immature erythrocytes are released from the bone marrow into the bloodstream. Macrocythemia, the presence of abnormally large erythrocytes, usually subsides after the first week of life and adult-sized red blood cells appear until the ninth week [11,34,35,36]. In our study, macrocythemia was present in all groups at birth without significant differences between groups. MCV significantly decreased in the fourth day of life in infants with IVH. Our findings are consistent with another study where MCV in EP infants was 111.11 ± 6.84 fL on the first day of life, decreasing to 102.88 ± 5.76 fL by 4–7 days [11].

The explanation provided is unclear but it could be related to the immature response of the hematopoietic system to chronic antenatal bleeding, pre/ intra-natal intraventricular or gastrointestinal bleeding, or infection-induced hemolysis. Severe intrauterine hypoxia secondary to pre-eclampsia, maternal/fetal thrombophilia, and maternal thrombocytopenia may cause fetal hemorrhages but most cases have no identifiable risk factors [37]. There are other intrauterine causes of prolonged intraventricular bleeding due to vasculitis, umbilical cord thrombosis, alterations in maternal/fetal blood pressure, maternal coagulopathy/use of anticoagulants or cocaine or methamphetamine abuse, maternal anemia, cholestasis of pregnancy, and neonatal alloimmune thrombocytopenia. Genetic factors such as proinflammatory interleukin Il-1ß or tumor necrosis factor genes, variants factor V Leiden, or prothrombin G20210A are also factors that influence fetal/neonatal bleeding involving vascular organization, inflammation, and coagulation disorders [37,38]. Our study did not consider maternal/fetal factors as previously described. Additionally, EP infants have lower iron stores at birth [39], a shorter lifespan of fetal erythrocytes, and lower bone marrow erythropoietic activity, which may not immediately compensate for intraventricular bleeding. During the first three days of life, erythrocytes may experience higher levels of oxidative damage, which can lead to functional and flexibility changes [40].

The mean cell hemoglobin (MCH) is the average amount of HGB per erythrocyte in the blood. In premature infants, MCH is typically higher (around 27–41 pg) than in term newborns [11]. Our research shows that EP infants without IVH had an MCH of 38.6 pg at birth. In contrast, infants with severe IVH had a significant reduction in their MCH levels on day 4 of life (35.6 pg) compared to those with mild IVH. This decline was linked to a sudden drop in HGB and HCT levels.

The mean corpuscular hemoglobin concentration (MCHC) is a measure of the amount of hemoglobin in each unit volume of red blood cells (hemoglobin × 100/hematocrit) and is usually consistent across various stages of gestational age. The MCHC reported values are typically between 31–34 g/dL and can be used as an indicator of anemia resulting from chronic or acute blood loss [36,39]. Our research has revealed that patients with severe IVH had a significantly lower MCHC due to substantial acute and chronic blood loss.

In our study, IVH did not affect the white blood cell lineage but we observed differences in the platelet lineage. A decrease in platelet count was observed in EP infants with IVH and severe IVH, even in the absence of thrombocytopenia, due to a consumptive process on DOL4. Other studies have also shown that a drop in platelet count during the first 7 days of life can predict IVH. [41,42]. We observed a significant increase in mean platelet volume (MPV) in patients with IVH and severe IVH on day 4, which was closely related to the decrease in platelet count. Previous studies have suggested that an elevated mean platelet volume (MPV) could indicate a risk for IVH. MPV can be used as an indicator of both platelet production and consumption. Increased platelet size indicates increased production preceded by consumption [43,44].

The secondary objective of our research was to review the neonatal risk factors associated with IVH in EP infants. The frequency of severe IVH was found to increase in association with the lack of antenatal corticosteroid prophylaxis, which is consistent with previous studies [45,46]. Infants delivered vaginally had a higher chance of developing IVH. This was confirmed by Gamaleldin and Humberg et al. [47,48]. We observed that hypercapnia, hypotension requiring treatment on the first day of life, and mechanical ventilation in the first four days of life were more common in the IVH group and severe IVH group. These findings are consistent with previous studies that have also supported this correlation [49,50,51]. Factors such as hypotension and hypercapnia can increase the risk of intraventricular hemorrhage (IVH) by affecting cerebral blood flow [52]. In our study, univariate regression analysis showed that infants with a gestational age of less than 26 weeks, a cord base excess (BE) of less than −8.23, an initial HCT level of less than 42.6%, and those who experienced hypercapnia and hypotension requiring treatment within the first few days of life were more likely to develop IVH, particularly severe IVH. A study found that a drop in hematocrit on day 2 of life increases the chance of severe IVH among EP infants weighing less than 750 g. [53]. A lack of antenatal dexamethasone, persistent ductus arteriosus, neonatal hypotension, and low HCT within the first 3 days of life was associated with severe IVH in very low birth weight neonates, according to a 2021 study by Al-Mouqdad [54].

Research should prioritize identifying measurable biomarkers, such as urine Activin A, S100B, and UCH-L1, to determine the risk of perinatal brain damage and IVH. Preventing these conditions is crucial in avoiding potential long-term developmental issues [55,56,57].

We need to acknowledge that our study has some limitations. Firstly, it is a retrospective study conducted at a single center and secondly, it involves a small population of EP infants with and without IVH. The population of EP infants is generally tiny and therefore, the small number of cases involved in the study could impact the statistical significance of the results. However, we want to mention that the statistical power of the analysis was significant and we utilized specific statistical techniques for small study groups to overcome this limitation. Larger sample sizes in multicenter trials should be considered to validate these findings further.

## 5. Conclusions

Extremely preterm infants with IVH may have low HCT and HGB at birth, followed by a further decrease in HCT, HGB, and PLT count by day four of life, accompanied by decreasing MCH and MCHC and increasing MPV. Gestational age, hypercapnia, hypotension, cord BE, and initial low HCT are the main neonatal factors associated with IVH. The predictive model based on logistic regression analysis can predict the likelihood of IVH in EP infants.

## Figures and Tables

**Table 1:** Distribution of IVH cases based on the day of occurrence.

Day of Life	Number of Cases	Percent of *n* = 134 (All Groups)	Percent of *n* = 48 (Cases with IVH)
DOL 1	14	10.45	29.2%
DOL 2	16	11.94	33.3%
DOL 3	8	5.97	16.7%
DOL 4	8	5.97	16.7%
DOL 5	2	1.50	4.2%
Without IVH	86	64.18	

**Table 2:** Comparison of the baseline, demographic, and clinical characteristics of EP infants according to IVH severity.

Clinical Characteristics	Control Group (without IVH) No = 86	Study Group with IVH (All Grades) *n* = 48	*p*-Value *	Mild IVH Subgroup (Grade 1–2) *n* = 30	Severe IVH Subgroup (Grade 3–4) *n* = 18	*p*-Value *
GA ^§^, weeks, median (quartile)	27 (25–28)	27 (26.5–28)	0.695	27 (27–28)	27 (24–28)	0.371
BW ^§^ (g), median (quartile)	870 (700–1000)	835 (650–975)	0.171	900 (800–980)	800 (600–820)	0.019 *
Male gender ^‡^, *n* (%)	32 (37.2)	30 (62.5)	0.004 *	20 (66.7)	10 (55.6)	0.441
SGA ^‡^, *n* (%)	14 (16.3)	14 (29.2)	0.083	6 (20)	8 (44.4)	0.073
No antenatal care ^‡^, *n* (%)	32 (37.2)	12 (25)	0.149	6 (20)	6 (33.3)	0.306
No ACT ^‡^, *n* (%)	38 (44.2)	30 (62.5)	0.041 *	14 (46.7)	16 (88.9)	0.002
Vaginal delivery ^‡^, *n* (%)	44 (51.2)	38 (79.2)	0.001 *	24 (80)	14 (77.8)	0.854
IVH, *n* (%)	-	48 (100)	-	-	-	-
grade 1	-	20 (41.7)	-	-	-	-
grade 2	-	10 (20.8)	-	-	-	-
grade 3	-	8 (16.7)	-	-	-	-
grade 4	-	10 (20.8)	-	-	-	-
Apgar 1 min ^§^, median (quartile)	7 (4–8)	5 (3–7)	0.043 *	6 (4–8)	5 (2–5)	0.007 *
Apgar 5 min ^§^, median (quartile)	8 (6–8)	7 (5–8)	0.115	8 (6–9)	5 (5–7)	0.004 *
DCC ^‡^, *n* (%)	20 (23.3)	3 (6.3)	0.0023 *	3 (10)	-	-
PBCC ^‡^, *n* (%)	39 (45.3)	25 (52.1)	0.0521	19 (63.3)	6 (33.3)	0.002 *
ICC ^‡^, *n* (%)	18 (20.9)	20 (41.7)	0.0267 *	8 (26.7)	12 (66.7)	0.001 *
RDS ^‡^, *n* (%)						
mild	10 (11.6)	0 (0)	0.018 *	0 (0)	0 (0)	0.238
moderate	38 (44.2)	18 (37.5)	0.021 *	14 (46.7)	4 (22.2)	0.014 *
severe	38 (44.2)	30 (62.5)	0.003 *	16 (53.3)	14 (77.8)	0.025 *
Surfactant ^‡^, *n* (%)	50 (58.1)	24 (50)	0.363	12 (40)	12 (66.7)	0.073
CPAP ^‡^, *n* (%)	60 (69.8)	16 (33.3)	<0.001 *	16 (53.3)	0 (0)	<0.001 *
MV ^‡^, *n* (%)	26 (30.2)	26 (54.2)	0.006 *	10 (33.3)	16 (88.9)	<0.001 *
HFOV ^‡^, *n* (%)	8 (9.3)	12 (25)	0.014 *	4 (13.3)	8 (44.4)	0.015 *
Hypercapnia ^‡^, *n* (%)	12 (13.9)	24 (50)	<0.001 *	8 (26.7)	16 (88.9)	<0.001 *
Hypotension ^‡^, *n* (%)	14 (16.3)	22 (45.8)	<0.001 *	6 (20)	16 (88.9)	<0.001 *
PDA ^‡^, *n* (%)	62 (72.1)	30 (62.5)	0.251	18 (60)	12 (66.7)	0.644
RCT ^‡^, days 4–7	-	20 (41.7)	-	2 (6.7)	18 (100)	<0.001 *
NICU ^§^, days, median (quartile)	21 (12–26)	17 (14–23)	0.298	20 (14–24)	15 (8–17)	0.002 *
Days at discharge ^§^, median (quartile)	50 (38–65)	36 (21–64)	0.008 *	52 (36–64)	23 (8–42)	0.011 *
Death ^‡^, *n* (%)	4 (4.7)	10 (20.8)	0.003	2 (6.7)	8 (44.4)	0.001 *

Continuous variables were expressed as median (quartile); the variables did not have a normal distribution; categorical variables: number (%); GA: gestational age; BW: birth weight; SGA: small for gestational age; ACT: antenatal corticosteroids therapy; PDA: patent ductus arteriosus; IVH: intraventricular hemorrhage; DCC: delayed cord clamping; PBCC: physiologic-based cord clamping; ICC: immediate cord clamping; RDS: respiratory distress syndrome; CPAP: continuous positive airway pressure; MV: mechanical ventilation; HFOV: high-frequency oscillatory ventilation; PDA: persistent ductus arteriosus; RCT: red cell transfusion; NICU: neonatal intensive care unit. § Mann–Whitney U test. ‡ Pearson Chi-square test. * Marked effects are significant at *p* < 0.05.

**Table 3:** Comparison of blood gases at birth and hematological parameters on the first and fourth day of life.

Blood Gases at Birth and Hematological Parameters	Control Group (without IVH) No = 86	Study Group with IVH (All Grades) *n* = 48	*p*-Value	Mild IVH Subgroup (Grade 1 and 2) *n* = 30	Severe IHV Subgroup (Grade 3 and 4) *n* = 18	*p*-Value
**At birth (cord blood gases), median (quartile)**						
pH	7.28 (7.22–7.33)	7.21 (7.09–7.27)	0.0008 *	7.26 (7.11–7.35)	7.15 (7.07–7.20)	0.0072 *
Base excess—BE, mmol/L	−4.9 (−7.5–−3.6)	−8.15 (−12.7–−5.9)	0.0066 *	−6 (−8.3–−5.6)	−10.6 (−12.7–−9.1)	0.0032 *
Lactate, mmol/L	2.6 (1.7–3.8)	4.7 (2.6–8.35)	0.0003 *	3 (2–8.1)	5.3 (3.9–8.6)	0.0187 *
**Red blood cell lineage, median (quartile)**						
HGB DOL1, g/dL	15.9 (13.9–16.5)	14.1 (13.4–15.1)	0.0312 *	14.7 (13.7–15.3)	13.1 (11.9–13.9)	0.0524
HGB DOL4^,^ g/dL	14.2 (12.7–14.5)	11.7 (9.6–12.3)	<0.001 *	12.4 (11.8–13.7)	7.3 (8.4–9.8)	<0.001 *
HGB difference between DOL1 and DOL4	1.7 (0.9–2.8)	2.45 (2.1–4)	<0.001 *	2.3 (2.2–3.6)	5.8 (2–6.5)	0.0029 *
HCT DOL1, %	51.2 (47.9–52.4)	43.8 (40.1–47.1)	0.0172 *	44.5 (42.2–46.5)	38.4 (37.1–42.6)	0.0561
HCT DOL4, %	45.1 (40.1–43.8)	35.2 (30.8–35.9)	<0.001 *	36.4 (34.8–37.8)	28.5 (25.4–29.8)	<0.001 *
HCT difference between DOL1 and DOL4	6.1 (5.9–7.3)	8.6 (7.3–10.6)	<0.001 *	8.1 (7.9–9.1)	9.9 (8.9–10.2)	0.0239 *
ERY DOL1, 10^6^/ µL	4.91 (3.82–5.34)	3.99 (3.34–4.52)	0.0251 *	4.21 (3.43–4.44)	3.84 (3.56–4.48)	0.0586
ERY DOL4, ×10^6^/ µL	3.97 (3.29–3.98)	3.02 (2.67–3.41)	<0.001 *	3.29 (2.67–3.82)	2.73 (2.46–02.92)	0.0093 *
ERY difference between DOL1 and DOL4	0.94 (0.72–1.18)	0.97 (0.83–1.21)	0.0122 *	0.92 (0.52–1.24)	1.11 (0.96–1.72)	0.0216 *
MCV DOL1, µm^3^	112.3 (109.7–116.4)	114.3 (108.8–118.5)	0.3729	116.3 (111.2–118.5)	110.1 (100.6–118.2)	0.1353
MCV DOL4, µm^3^	105.4 (100.8–109.1)	102.9 (95.4–108.1)	0.0278 *	104.9 (94.6–108.8)	100 (96.2–102.2)	0.2464
MCV difference between DOL1 and DOL4	7.5 (2.4–11.9)	8.35 (6.05–16.25)	0.0146 *	8.4 (7.5–17.2)	8.1 (4.4–15.3)	0.3943
MCH DOL1, pg	38.6 (37–39.5)	31.8 (36.3–40.1)	0.1875	39.2 (37.9–40.5)	35.6 (34.3–36.9)	<0.001 *
MCH DOL4, pg	36.7 (34.8–40.1)	34.2 (31.6–37.3)	<0.001 *	35.2 (31.8–39.2)	31.6 (30.7–33.6)	0.0005 *
MCH difference between DOL1 and DOL4	1.4 (−2–3.2)	3.7 (1.9–5.7)	0.0002 *	3.2 (−0.2–5.8)	4 (2.5–4)	0.7982
MCHC DOL1, g/dL	34 (33.6–34.9)	34.1 (32.4–34.6)	0.0717	34.3 (33.3–34.8)	32.4 (32.2–34.1)	0.0041 *
MCHC DOL4, g/dL	34.8 (33.5–36.3)	34.7 (34.1–35.3)	0.3158	35.1 (34.3–35.9)	33.9 (33.4–34.3)	0.0001 *
MCHC difference between DOL1 and DOL4	−0.3 (−2.1–0.30)	−1 (−1.9–0.1)	0.5576	−1 (−2–0)	−0.2 (−1.9–0.2)	0.3272
**White blood cell lineage, median (quartile)**						
WBC DOL1, ×10^3^/µL	13.63 (10.3–22.38)	15.09 (9.19–21.07)	0.5713	17.9 (13.63–20.2)	14.56 (6.3–21.94)	0.2167
WBC DOL4, ×10^3^/µL	13.26 (9.9–16.47)	12.68 (11.4–16.9)	0.5401	12.67 (9.8–16.9)	16 (11.4–17.5)	0.1867
WBC difference between DOL1 and DOL4	1.5 (−2.53–5.48)	2.16 (−2.27–6.72)	0.5525	2.16 (1–7.5)	−2.94 (−5.1–4.9)	0.0191 *
ANC DOL1, ×10^3^/µL	5.5 (2.9–9.5)	7.4 (3.4–9.3)	0.2536	7.8 (3.83–10.6)	4.3 (3.06–8.31)	0.0152 *
ANC DOL4, ×10^3^/µL	4.53 (2.97–7.7)	5 (2.25–7.73)	0.8382	5.62 (2.35–7.990	4.3 (1.8–5)	0.0332 *
ANC difference between DOL1 and DOL 4	0.6 (−2.14–3.57)	1.37 (0.11–2.05)	0.2734	1.48 (−0.08–2.6)	1.26 (1.21–1.9)	0.9660
I/T ratio DOL1	0.14 (0.09–0.19)	0.13 (0.09–018)	0.3485	0.14 (0.1–0.17)	0.10 (0.06–0.18)	0.3710
I/T ratio DOL4	0.18 (0.12–0.20)	0.14 (0.07–0.18)	0.0007	0.10 (0.07–0.17)	0.14 (0.12–0.18)	0.2501
I/T ratio difference between DOL1 and DOL4	−0.04 (−0.06–0.04)	0.01 (−004–0.08)	0.0042	0.02 (−0.04–0.10)	−0.01 (−0.04–0.06)	0.036 8 *
**Platelet lineage, median (quartile)**						
PLT DOL1, ×10^3^/µL	210 (178–272)	203 (153–302)	0.6722	274 (161–346)	184 (145–247)	0.0609
PLT DOL4, ×10^3^/µL	258 (178–320)	201 (132–279)	0.0098 *	256 (113–331)	166 (134–174)	0.0106 *
PLT difference between DOL1 and DOL 4	−27 (−85–22)	43.5 (−17.5–65)	0.0004 *	29 (−75–59)	50 (2–113)	0.0966
MPV DOL1, μm^3^	10.1 (9.7–10.7)	10.1 (9.5–11.2)	0.9334	9.7 (9.4–11.2)	10.4 (9.9–11.4)	0.0752
MPV DOL4, μm^3^	10.7 (10.3–11.3)	11.3 (10.9–11.8)	0.0009 *	11.3 (11–12.2)	10.9 (10.5–11.8)	0.1729
MPV difference between DOL1 and DOL4	−0.6 (−1–−0.3)	−1.3 (−1.6–−0.4)	0.0139	−1.4 (−1.8–−0.9)	−0.4 (−1.4–0)	0.0413
PCT DOL1, %	0.22 (0.19–0.27)	0.20 (0.15–0.32)	0.3931	0.26 (0.18–0.33)	0.19 (0.13–0.23)	0.1182
PCT DOL4^,^ %	0.27 (0.20–0.32)	0.28 (0.22–0.32)	0.8623	0.28 (0.14–0.34)	0.27 (0.22–0.31)	0.8313
PCT difference between DOL1 and DOL4	−0.04 (−0.1–0.03)	−0.02 (−0.09–0.02)	0.6994	−0.01 (−0.09–0.04)	−0.04 (−0.12–0)	0.0991

IVH: intraventricular hemorrhage; DOL1: Birth; DOL4: the fourth day of life; HGB: hemoglobin concentration; HCT: hematocrit; ERY: erythrocytes; MCV: mean corpuscular volume; MCH: mean corpuscular hemoglobin; MCHC: mean corpuscular hemoglobin concentration; WBC: white blood cells; ANC: absolute neutrophil count; I/T: immature to total leukocyte ratio; PLT: platelet count; MPV: mean platelet volume; PCT: plateletcrit. * Marked effects are significant at *p* < 0.05.

**Table 4:** IVH risk assessment in EP infants—neonatal factors.

Univariate Analysis	AUC (95% Confidence Interval)	Std. Error	*p*-Value	Se	Sp	Cut-Off Levels
GA, weeks	0.760 (0.669–0.851)	0.046	0.001 *	86.5	83.4	26
Apgar 1 min	0.606 (0.508–0.703)	0.050	0.043	78.4	68.7	5
Apgar 5 min	0.582 (0.482–0.683)	0.051	0.115	65.3	54.7	6
MV, yes	0.620 (0.519–0.720)	0.051	0.022 *	82.9	81.6	-
HFOV, yes	0.578 (0.474–0.683)	0.053	0.133	62.1	50.3	-
Hypercapnia, yes	0.680 (0.581–0.780)	0.051	0.001 *	87.1	82.8	-
Hypotension, yes	0.648 (0.547–0.749)	0.052	0.005 *	75.6	77.5	-
pH	0.673 (0.575–0.772)	0.050	0.001 *	55.8	52.9	7.09
Base excess—BE, mmol/L	0.742 (0.656–0.829)	0.044	<0.001 *	68.2	77.8	−8.23
Lactate, mmol/L	0.686 (0.588–0.783)	0.050	<0.001 *	74.1	69.4	4.8
HGB DOL1, g/dL	0.806 (0.721–0.958)	0.023	0.002 *	89.6	88.6	13.9
HCT DOL1, %	0.922 (0.841–0.985)	0.019	<0.001 *	88.4	91.3	42.6
ERY DOL1, 10^6^/µL	0.895 (0.796–0.963)	0.035	0.001 *	89.6	90.4	3.82

IVH: intraventricular hemorrhage; GA: gestational age; MV: mechanical ventilation; HFOV: high-frequency oscillatory ventilation; DOL1: birth; DOL4: the fourth day of life; HGB: hemoglobin concentration; HCT: hematocrit; ERY: erythrocytes; AUC—area under the curve; Se—sensitivity; Sp—specificity; Std. error–standard error; * Marked effects are significant at *p* < 0.05.

**Table 5:** Multivariate analysis (multiple regression—logistic regression model) of the association of neonatal parameters with IVH.

	β	S.E.	*p*-Value	OR Exp(B)	95% CI for EXP(B)	
					Lower	Upper
**Neonatal Parameters**						
Gestational age	β_1_: 3.002	0.002	0.017 *	3.002	2.684	4.236
Hypercapnia	β_2_: 1.564	0.506	0.002 *	4.780	2.774	4.879
Hypotension	β_3_: 1.581	0.670	0.018 *	4.860	2.308	5.062
BE	β_4_: −0.250	0.106	0.018 *	1.779	1.634	2.958
Constant	−5.572	2.161	0.010 *	0.004		

OR odds ratio; CI, confidence interval. Crude OR was estimated by logistic regression. * Marked effects are significant at *p* < 0.05.

## Data Availability

The data presented in this study are available on request from the corresponding author.
